# Demonstration of electric micropropulsion multimodality

**DOI:** 10.1126/sciadv.adc9850

**Published:** 2022-09-07

**Authors:** Denis B. Zolotukhin, Siva Ram Prasad Bandaru, Keir P. Daniels, Isak I. Beilis, Michael Keidar

**Affiliations:** ^1^ George Washington University, 800 22nd Street Northwest, Washington, DC 20052, USA.; ^2^ Tomsk State University of Control Systems and Radioelectronics, 40 Lenin Ave., Tomsk 634050, Russia.; ^3^ Department of Electrical Engineering, Tel Aviv University, Tel Aviv, Israel.

## Abstract

Electric propulsion has become popular nowadays owing to the trend of miniaturizing the size and mass of satellites. However, the main drawback of the most popular approach—Hall thrusters—is that their efficiency and thrust-to-power ratio (TPR) markedly deteriorate when its size and power level are reduced. Here, we demonstrate an alternative approach—a minute low-power (<50 W), lightweight (~100 g), two-stage propulsion system. The system is based on a micro-cathode vacuum arc thruster with magnetoplasmadynamic second stage (μCAT-MPD), which achieves the following parameters: a thrust of up to 1.7 mN at a TPR of 37 μN/W and an efficiency of ~50%. A μCAT-MPD system, in addition to “traditional” inverse, displays the anomalous direct (growing) “TPR versus specific impulse *I*_sp_” trend at high *I*_sp_ values and allows multimodality at high efficiency.

## Teaser

A two-stage low-power vacuum arc MPD thruster achieves effective performance with multimodality in operational regimes.

## INTRODUCTION

Further progress in space satellite technologies is highly associated with a decrease in satellites’ launch cost, which is directly proportional to satellite mass and size. This has led to the miniaturization of satellites as a design trend in the space industry (*1*). Minute electric propulsion subsystems seem to be the most promising, since they use electromagnetic plasma acceleration to create high exhaust speed (therefore, high efficiency with less propellant storage onboard), and they also provide a convenient way to control the thruster parameters over a wide range by varying the electrical power level (*2*). One of the most mature and widely used electric propulsion technologies is the Hall thruster, which uses xenon as a propellant. Although such thrusters operate at low (60 to 270 W) power suitable for propulsion of small satellites, they provide a sufficient range of thrusts (from 5 to 25 mN) at a specific impulse of about 400 to 1800 s and a high thrust-to-power ratio (TPR) of about 65 μN/W (*3*). However, xenon is a very expensive rare noble gas with a worldwide production limited to only about 50 to 60 tons (*4*), and therefore, xenon-based electric propulsion has to compete with other industries [imaging and lighting, electronics and semiconductor industry (*5*), medicine (*6*), etc.] for xenon consumption. A promising electric propulsion design for small satellites is the micro-cathode vacuum arc thrusters (μCATs). Their key advantage is that they use a propellant stored as a solid—a state of matter with the highest atomic density, which can be stored much more efficiently than a liquid or gaseous propellant. Its solid electrodes can be made of widespread and relatively cheap metals such as copper or titanium. During the μCAT firing, a solid converts into plasma, which can be further accelerated by electromagnetic fields. Despite the other miniature electrical thrusters with a solid propellant (such as an iodine thruster) (*7*), μCATs do not use toxic propellants, and they do not have a warm-up time; i.e., they can be ignited instantly after providing the respective control signal. The μCATs’ performance has been successfully demonstrated in flight tests during Ballistic ReInforced Communication Satellite (BRICSat), The CubeSat Astronomy by NASA and Yonsei using the Virtual Telescope ALignment-eXperiment (CANYVAL-X), and Ballistic ReInforced Communication Satellite–Propulsive Test Unit (BRICSat-P) missions (*8*–*10*). Typical thrust *T* and specific impulse *I*_sp_ levels for μCATs are within 50 μN and 3000 s, with a TPR of less than 4 μN/W at 1- to 15-W power (*10*). The challenge for this design is that, for some applications, such as orbit raising maneuvers, a much higher thrust value is needed. In addition, the power and propellant storage limitations on small satellites require higher values of TPR and energetic efficiency. Recent tests on design modifications of this thruster (*11*) demonstrated that adding a second magnetoplasmadynamic (MPD) stage to μCAT allows the thruster to simultaneously achieve higher levels of thrust (~210 μN), TPR (~18 μN/W), and efficiency (~50%). Although it was found (*11*) that, after the onset of the magnetized vacuum arc, the performance of the two-stage μCAT-MPD thruster rapidly jumps, it was still unclear how will the performance behave with the further increase of the power delivered to the second stage. The presence of residual plasma after the first stage leads to cathode spot formation when the second-stage voltage is applied. Such cathode spots have different characteristics. Slow-moving spots appear during arc development (100 μs and more), when the cathode is heated locally, and in the near-cathode region, a dense plasma exists, impeding the cathode plasma flow (*12*, *13*). The effect of the increased mass flow rate due to much more intense ion generation in a powerful second-stage discharge with simultaneous ion acceleration by the Lorentz force on the specific impulse of the device has also remained unexplored. The question regarding the accessibility of higher thrust, TPR, and efficiency as well as the relationships between *I*_sp_ and TPR for this two-stage μCAT-MPD thruster was beyond the scope of the previous research. We address these critical issues in the present paper.

## RESULTS

**Fig. 1.**
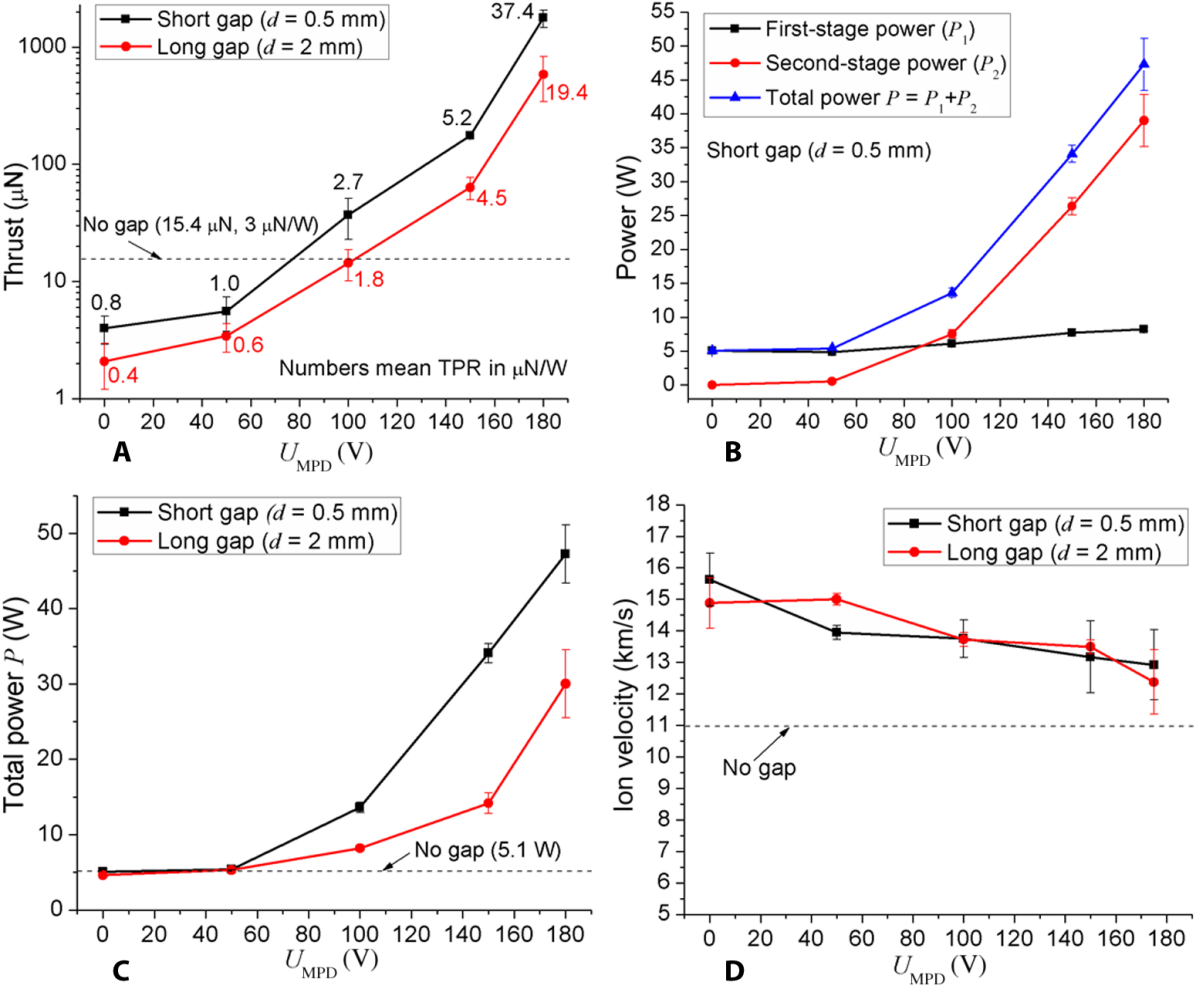
Experimental results of the μCAT-MPD thruster performance characterization. Thrust (**A**), power in both stages for the short gap (**B**), total power for the long and short gaps (**C**), and ion velocity (**D**) versus the second-stage (MPD) voltage *U*_MPD_. Ti cathode, Cathode material is titanium.

**Fig. 2.**
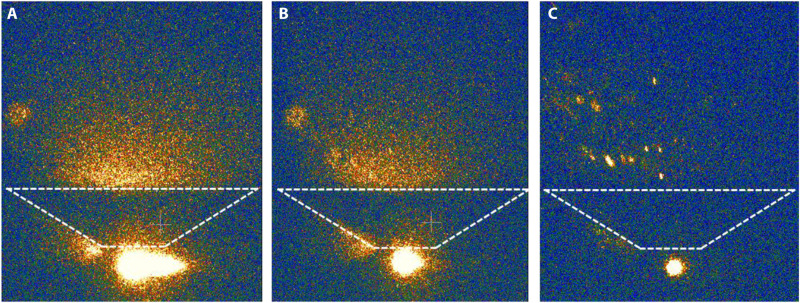
Images of the plasma plume captured at different moments. (**A**) the moment of the maximal current in the first stage *t*_max_, (**B**) *t*_max_ + 240 μs, (**C**) *t*_max_ + 370 μs.

The developed design of the μCAT-MPD thruster allowed achieving the performance parameters that are superior to many known propulsion devices operating at comparable power levels (Fig. 1). Figure 1A demonstrates that the gap between the first and second stages affects the thrust level: A lower gap generates higher thrust values, while a zero gap (when the second-stage electrode becomes the part of the anode and acquires its potential) generates a fixed value of ~15 μN and cannot be throttled. At the optimal (short) gap, the thrust incrementally grows (within three orders of magnitude) with the total power *P* = *P*_1_ + *P*_2_ determined by *U*_MPD_ and lastly reaches 1.7 mN. Note that the thrust growth rate with *U*_MPD_ does not show any saturation at high *U*_MPD_: Thus, it appears physically possible to have higher thrust values if the second-stage voltage is increased further. Note also that the thrust grows faster than the power; thus, TPR also grows incrementally and reaches 37.4 μN/W (in the case of a short gap). The power dissipated in both stages of the thruster is given in Fig. 1 (B and C). Extraction of high ionic currents with activated second stage leads to only moderate increase of the power dissipated in the first stage, while the first-stage discharge burning voltage grows mainly owing to the magnetization of the plasma electrons (*11*). We can see from Fig. 1B that both—the first and the second stages—powers grow with *U*_MPD_; however, this trend is much stronger for *P*_2_ rather than for *P*_1_. Despite this growth, the total power *P* remains relatively low—below 50 W (Fig. 1C), which indicates that such a thruster can still be considered as a low-power device. Figure 1D demonstrates that for any nonzero gap between the thruster stages, the exhausting ion velocity does not change notable with the increase in the *U*_MPD_ voltage. This indicates that the specific impulse *I*_sp_ of the thruster does not degrade significantly with a proportional growth in thrust with the *U*_MPD_. Since the thruster demonstrates the highest performance with a short gap (Fig. 1A), the experimental results below are given for the “short gap” case only. The photos obtained by an intensified charge-coupled device camera (Fig. 2) demonstrate that plasma plume during the discharge firing is directed from the first-stage cathode toward the thruster exhaust mostly inside the volume of the cone, not from its side.

**Fig. 3.**
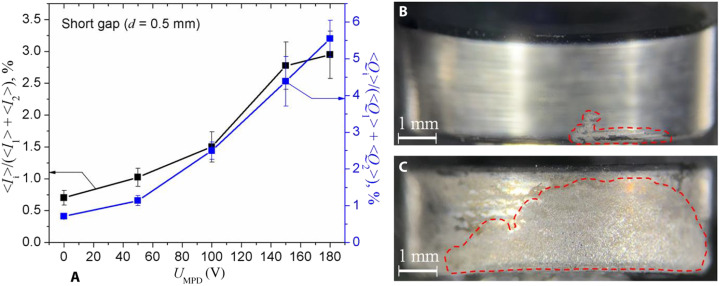
Results demonstrating the change in thruster operation with the activation of the second stage. The ratios of pulse-averaged total current (left axis) and charge (right axis) of exhausting ions by the thruster to the respective sums of pulse-averaged discharge currents and charges in both stages (**A**). Images of the side surfaces of the cathodes after firing of the first stage only (**B**) and together with the second stage (**C**) within the single pulse. The image in (B) outside the dashed region was blurred to focus the attention on the significant details of the surface; the original image can be seen in fig. S3.

The fractions of pulse-averaged total current 〈*I*_i_〉 and charge 〈*Q*_i_〉 of ions expelled by the thruster (Fig. 3A) demonstrate that the thruster produces and expels more ions for the same amount of discharge currents in both stages with the increase of *U*_MPD_. Images of the cathodes with traces of the cathodic spots (Fig. 3, B and C) demonstrate that the activation of discharge in the second stage leads to significant expansion of the erosion area in comparison with the case when the plasma is produced by the first stage only.

**Fig. 4.**
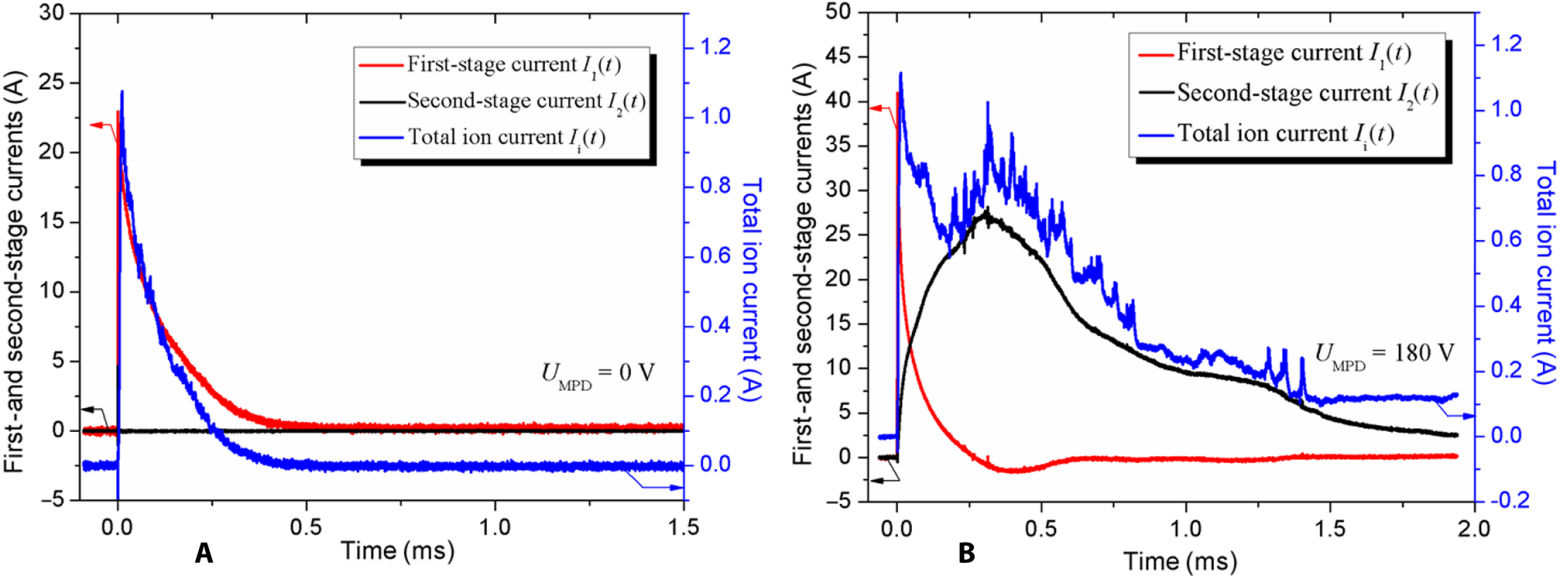
Temporal characteristics of the first- and second-stage currents as well as the total currents of exhausting ions for the two-stage μCAT-MPD thruster. Cases with (**A**) zero and (**B**) maximal (*U*_MPD_ = 180 V) voltage on the second stage.

Results in Fig. 3 (B and C) indicate that the preliminary plasma, produced by the first stage from a tiny area occupied by cathode spots, leads to the formation of the large-area cathode spots after supplying second-stage voltage. The cathode spot area, shown in Fig. 3B, was produced by a relatively short arc pulse (tens of microseconds), while the arc duration of the second-stage arc (shown in Fig. 3C) is significantly larger (up to several milliseconds) at a pulse-averaged current of up to 9 A. Taking into account that the arc at the second stage was ignited already at the background of the relatively dense plasma (~10^20^ m^3^) generated at the first stage, the observed change of the cathode spot areas can be understood as a result of change in the dynamics of spot behavior and their types. According to well-known investigations (*12*), the arc current, arc duration, surrounding plasma (gas) pressure, and cathode surface state determine the different spot types, which are characterized by spot lifetime, velocity, spot current, and cathode erosion. It was established that spot type changed from fast- to slow-moving spots with the arc time duration (*12*). At the arc beginning, the fast- and short-lived (<10 μs) spots were observed. These cathode spots typically appear at a thin oxide or impurity films and lead to a relatively low cathode erosion rate (*13*). On the other hand, the slow-moving spots appear during arc development (100 μs and more), when the cathode is heated locally, and in the near-cathode region, a dense plasma exists, impeding the cathode plasma flow. As a result, the cathode erosion rate significantly increases with respect to that rate produced by fast spots. The influence of the background plasma with a density of ~10^20^ m^3^ on the time of development of the cathode plasma plume was also observed for a much longer arc time—from 1 s to a few tens of seconds for different cathode materials (*14*). Kimblin (*15*) also experimentally demonstrated the relatively large fraction of ion current for long-duration arcs in the range of 0.1 to 3 s. These processes explain the increase of the cathode erosion (flow rate) and, consequently, the thrust-to-power ratio in the case of a two-stage thruster configuration. Aforementioned features of the cathodic spots in the single- and two-stage firing regimes manifest themselves into different waveforms of the first- and second-stage currents, resulting in the change of the total ion current waveform (Fig. 4). With the zero second-stage voltage (*U*_MPD_ = 0 V; Fig. 4A), no current is flowing in the second stage, and the total ion current is short and mimics the waveform of the first-stage current. High second-stage voltage (*U*_MPD_ = 180 V; Fig. 4A) initiates longer second-stage current pulse, which results in high amplitude and duration of the total ion current in which waveform now looks like a superposition of the waveforms of both discharge currents.

**Fig. 5.**
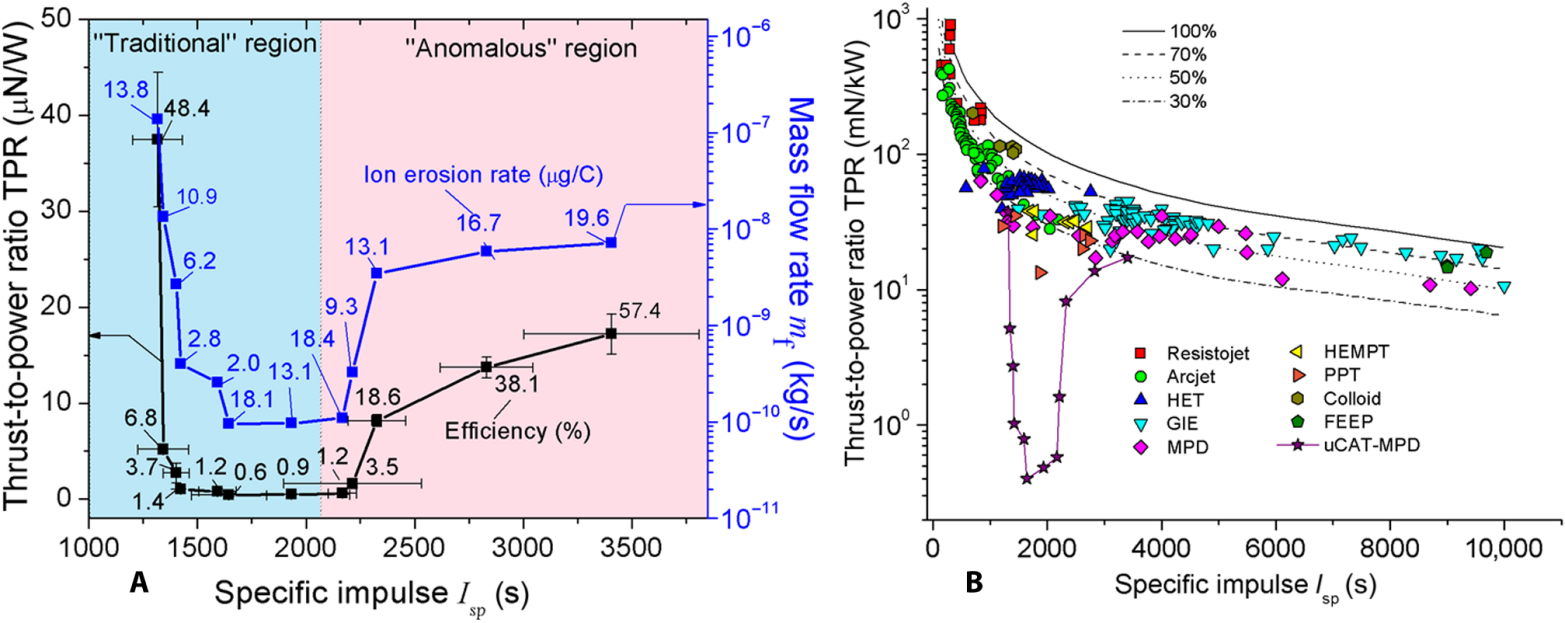
Unusual trends in the thruster performance. The trends of TPR (left axis) and mass flow rate (right axis) versus the *I*_sp_ for the two-stage μCAT-MPD thruster, with the planar electrodes, movable magnet, and the short gap between the first and second stages (**A**). For comparison, TPR versus *I*_sp_ trends (**B**) for different approaches of the powerful thrusters (*32*–*34*): HET, Hall effect thruster; GIE, gridded ion engine; MPD, magnetoplasmadynamic thruster; HEMPT, high-efficiency multistage plasma thruster; PPT, pulsing plasma thruster; FEEP, field-emission electric propulsion; and μCAT-MPD, micro-cathode arc thruster with MPD stage (this work). Black curves mean the trends for the fixed values of the efficiency (100% − 30%).

A more detailed study of the interconnection between TPR and *I*_sp_ for the thruster with variable position of magnet with respect to the discharge area has revealed interesting nonmonotonous connections between these parameters (Fig. 5A). One can see (Fig. 5A) that the TPR versus *I*_sp_ trend has two characteristic regions: decreasing (blue region) and increasing (pink region). In the decreasing part, the highest TPR of 37 μN/W is observed for the highest values of voltage *U*_MPD_ = 180 V and the total power *P* = 48.8 W. This decreasing part of TPR versus *I*_sp_ trend can be called “traditional,” since it is typical for most of the propulsion devices working within a broad range of thrust, TPR, and power levels (Fig. 5B). The other (“anomalous”) region at higher *I*_sp_, which has the growing TPR versus *I*_sp_ trend, has not been observed before for any other device in the literature (Fig. 5B). Comparison of “TPR versus *I*_sp_” with “mass flow rate versus *I*_sp_” trends provides additional argument to support the claim that the main physical reason for the growth of TPR is the increase of mass flow rate at the same power: These two trends match each other (Fig. 5A).

Although the problem of production of macroparticles in vacuum arc discharges is well known, these particles do not contribute significantly to the generated thrust due to the following reasons: Ion erosion rate *E*_r_, which we estimated as $E_{r}=\alpha_{i}M_{i}/e\bar{Z}$ [where α_i_ = *Q*_i_/(*Q*_1_ + *Q*_2_) is the normalized integral of total ion current, *M*_i_ is the atomic mass of cathode material, *e* is the elementary charge, and $\bar{Z}$ is the mean charge state of the ion] and plotted in Fig. 5A, was found to be slightly lower than or comparable with the erosion rates from the literature for Ti (22.4 μg/C) or Cu (33.4 μg/C) cathodes (*16*). Accounting for the microparticles may increase the cathode erosion rate; however, as follows from Daalder (*17*), their production becomes noticeable only after reaching the high values (about 1 C) of the discharge current integral at high current amplitudes (around hundreds of amperes) and pulse durations (around seconds), which is not the case for our thruster. In addition, magnetic field causes the motion of the cathodic spot over the cathode surface, which, in turn, decreases the local thermal loads on the cathode and lastly minimizes the generation of macroparticles (*18*). Moreover, these macroparticles typically have much lower velocities (10^2^ to 10^3^ m/s) (*19*–*21*) than ions (several kilometers per second). As such, it is not expected that macroparticles can contribute to thrust.

## DISCUSSION

**Fig. 6.**
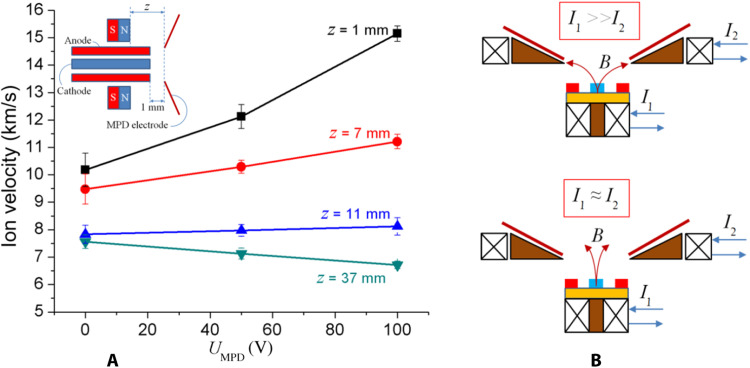
Acceleration of ions and the way to control it. Ion velocity for the two-stage μCAT-MPD thruster with the cylindrical copper cathode and anode versus the distance *z* between the magnet face and the opening of the second-stage MPD electrode (**A**); sketch demonstrating the possibility to control the modulus and direction of the magnetic field strength by the two independent electromagnetic coils with cores (**B**).

The presence of traditional and anomalous TPR versus *I*_sp_ trends can be explained by the fact that the exhausting ion velocity of the μCAT-MPD thruster depends on the magnetic field strength and configuration. In the case of permanent magnet, it can be regulated by the position *z* of the magnet with respect to the second stage (Fig. 6A). Figure 6A demonstrates that, when *z* is short, the radial component of the magnetic field *B*_r_ reaches a value high enough for a significant contribution of Lorentz force to ion acceleration, and therefore, the ion velocity grows with the second-stage voltage *U*_MPD_. However, increasing the distance between the magnet and the second stage results in a more moderate growth of ion velocities, and lastly, when the magnet is far enough (*z* = 37 mm), the ions start to decelerate due to the positive second-stage potential, with the second-stage discharge attempting to switch into uncontrollable dc mode.

One can see that, generally, ion velocities in Fig. 6A are lower than in Fig. 2D. This difference comes from the cathode material: The ions ejected from the titanium cathode (Fig. 2D) typically have narrow velocity distribution, with a peak around 17 km/s and a high-velocity tail up to 24 km/s (*22*), while the copper cathode (Fig. 6A) expels heavier ions with quite broad distribution of initial ion velocities from 9 km/s with the peak around 13 to 15 km/s (*22*, *23*). Nevertheless, the data in Fig. 6A illustrate the effect of the magnetic field on the ions’ acceleration: Even at *U*_MPD_ = 0, the ion velocity is higher when the magnet is located closer to the discharge area, justifying that the ions accelerate due to the magnetic mirror effect (*23*, *24*). An additional source of ion acceleration comes from the Lorentz force *F*_L_ ~ *j*_θ_ × *B*_r_, which contributes to thrust at higher values of the magnetic field in the vicinity of the discharge region and discharge currents (and, correspondingly, electron currents in plasma). Previous simulations of the contribution of *F*_L_ to thrust (*11*) showed that the *j*_θ_ × *B*_r_ acceleration region has a typical length of within 1 mm; thus, the contribution of Lorentz force to thrust can be comparable with the initial thrust (*25*) and strongly depends on the magnetic field strength and configuration defined by the position of the magnet with respect to the discharge area.

These results demonstrate that such a two-stage μCAT-MPD thruster has another independent “knob” for controlling its performance, namely, the position of the magnet with respect to the discharge region. For the on-orbit application of the thruster, instead of moving the permanent magnet, a more practical approach can be implemented for the change of the magnetic field strength and configuration—by varying the absolute values and relative ratios of the currents *I*_1_ and *I*_2_ feeding two independent electromagnetic coils with cores (Fig. 6B).

Low-power vacuum arc thrusters have the known problem of a limited lifetime, which may vary from 10^3^ (*26*) to 10^5^ pulses (*27*), while the special constructions with the cathode feeding mechanism allow the achievement of several million pulses (*28*). In our previous study (*29*), we found that the lifetime of the μCAT firing in the high-power regime can be extended to more than 1 million pulses by optimizing the anode-cathode gap (extending it to around 1 mm) and providing the magnetic field, causing the movement of cathodic spot along the cathode surface. Thus, the configuration of the first-stage electrodes of the newly designed μCAT-MPD thruster was chosen to provide the maximal lifetime even at a high-power performance.

## MATERIALS AND METHODS

**Fig. 7.**
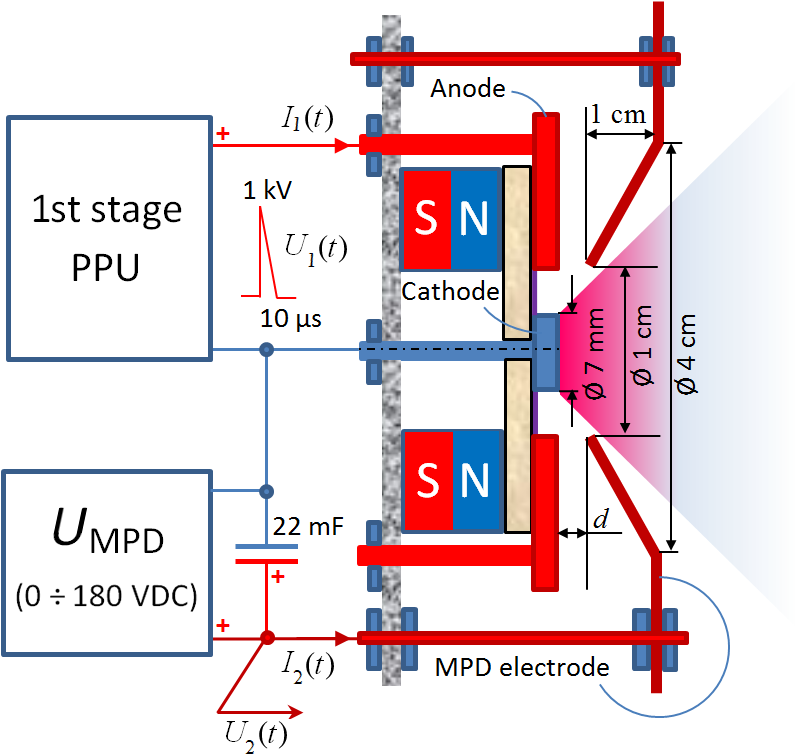
Schematic of the μCAT-MPD thruster. Preliminary plasma, generated by an arc discharge between cathode and anode of the first stage, initiates a more powerful arc discharge between the second-stage conical MPD electrode and the cathode. Then, the interaction of the azimuthal electronic current in the second-stage plasma and the radial component of magnetic field accelerates the plasma and generates the thrust.

Schematic view of the developed thruster is shown in Fig. 7. The first stage (a single μCAT) had a planar configuration, with a central disc-shaped Ti cathode and an outer ring-shaped Cu anode, which were both placed on a disc-shaped ceramic washer. A ring-shaped (1.27 cm inner diameter, 2.54 mm outer diameter, and 1.27 cm length) permanent axially magnetized magnet with an induction of ~0.1 T on its axis was placed behind the ceramic washer, providing thermal isolation from the first stage. The ceramic washer surface of the first-stage anode-cathode gap was covered by weakly conductive carbon paint, ensuring the initiation of a triggerless (*30*) arc discharge with the first-stage pulse duration τ_1_, current *I*_1_(*t*), voltage *U*_1_(*t*), and the average power *P*_1_. As a second stage, a conical frustum (made of thin molybdenum foil with a narrow opening separated by the gap *d* from the cathode surface) was used. Preliminary plasma from the first stage, due to the dc voltage *U*_MPD_ between the cathode and the second-stage electrode, initiated a more powerful arc discharge in the second stage with the second-stage pulse duration τ_2_, current *I*_2_(*t*), voltage *U*_2_(*t*), and the average power *P*_2_. The interaction of azimuthal electronic current *j*_θ_, flowing in plasma, with the radial component of the external magnetic field *B*_r_ resulted in the formation of the Lorentz force *F*_L_ ~ *j*_θ_ × *B*_r_, accelerating the plasma toward the wide opening of the MPD electrode and enhancing the thrust. More information on thruster performance, characterization, and thrust measurements can be found in Supplementary Materials and Methods.

To conclude, the newly designed configuration of the μCAT-MPD thruster achieves a superior combination of performance parameters: thrust (up to 1.7 mN) together with TPR (up to 37 μN/W), consuming electrical power below 50 W and efficiency (up to 57%). The TPR values achieved are superior for low-power devices used for electric propulsion on small satellites and even comparable to the values for more powerful and mature technologies (Fig. 6). Such advantages of vacuum arcs as the almost 100% degree of ionization in plasmas, as well as the very nature of the cathodic spot having almost infinite emission of the charged particles (*31*), speak to the expectation of even higher values of performance parameters of such devices that may be achieved with the future progress of their technology.

## Supplementary Materials

### This PDF file includes:

Supplementary Materials and Methods Figs. S1 to S3 References
