# Supplementary material for: Demonstration of electric micropropulsion multimodality
Source: Sci Adv. 2022 Sep 7;8(36):eadc9850. doi: 10.1126/sciadv.adc9850 (PMC9451150; doi:10.1126/sciadv.adc9850)
Supplement: Supplementary file 1 — Supplementary Materials and Methods Figs. S1 to S3 References [file sciadv.adc9850_sm.pdf]

Science Advances
AAAS

Supplementary Materials for

Demonstration of electric micropropulsion multimodality

Denis B. Zolotukhin *et al.*

Corresponding author: Denis B. Zolotukhin, zolotukhinden@gmail.com;
Michael Keidar, keidar@gwu.edu

Sci. Adv. **8**, eadc9850 (2022)
DOI: 10.1126/sciadv.adc9850

This PDF file includes:

Supplementary Materials and Methods
Figs. S1 to S3
References

Supplementary Materials and Methods

Characterization of discharge characteristics of the thruster

A more detailed electrical circuit of the thruster is given in the Fig. S1.

The first-stage power processing unit (PPU) consisted of a 25 V dc power supply (BK Precision 1550), a polar capacitor C_1 = 2.2 mF, an inductor L = 550 µH with ferrite core, and an IGBT (IXYH50N120C3). After applying to the IGBT gate a control rectangular signal (with amplitude of 5 V, 0.35 ms pulse width and variable pulse repetition rate f up to 20 Hz), the first-stage PPU formed a peak of voltage with amplitude of up to 1200 V and a duration of several tens of micro-seconds. This voltage, applied between anode and cathode of the thruster, resulted in the formation of the preliminary arc discharge plasma with the first-stage discharge voltage $U_1(t)$ (measured by Tektronix P6015 high-voltage probe) and discharge current $I_1(t)$ (measured by Pearson model 110 current monitor). The second-stage discharge with voltage $U_2(t)$ (measured by Tektronix P2220 probe) and current $I_2(t)$ (measured by Pearson model 110 current monitor) is ignited due to the dc U_{MPD} voltage (up to 180 V, stabilized by a polar capacitor C_2 = 10.7 mF) applied between the cathode and the second-stage MPD electrode. All waveforms of current and voltage were displayed, digitized and stored for further processing after averaging over 256 single waveforms using a digital TDS3014C oscilloscope.

Average powers dissipated in the first P_1 and the second P_2 stages of the thruster were calculated using the formulas:

$$P_1 = \int_0^{\tau_1} I_1(t)U_1(t)dt, \quad (1)$$

$$P_2 = \int_0^{\tau_2} I_2(t)U_2(t)dt, \quad (2)$$

where τ_1, τ_1 are the pulse widths of the first- and second-stage instantaneous powers. The errors for P_1, P_2 were estimated as standard deviations $\delta(P_1)$, $\delta(P_2)$ over up to 6 experimental trials.

The total power P dissipated in the both stages within single experimental trial was estimated by summarizing P_1 and P_2:

$$P = P_1 + P_2 \quad (3)$$

The error for P was estimated using the standard deviations $\delta(P_1)$, $\delta(P_2)$ for the first and second-stage powers:

$$\delta(P) = \sqrt{\delta^2(P_1) + \delta^2(P_2)}, \quad (4)$$

The final value of the total power $\langle P \rangle$ was obtained as the average value of all experimental total powers P obtained at the same experimental conditions.

Thrust and thrust-to-power ratio

The average thrust was measured by placing the thruster directly on the movable arm of the torsional thrust stand (see the Fig. S2).

Thruster firing caused the deflection of the movable arm around its axis of rotation; this deflection was measured by the laser sensor. Using the set of fins placed on high-precision scale (identical to the fins placed on the end of the thrust stand arm), this deflection was recalculated to a force (i.e. thrust). More detailed information regarding thrust stand measurements are given in our previous work (*25*). The final value of the average thrust $\langle T \rangle$ then was estimated as the

average value of thrusts *T* obtained within up to 6 experimental trials at the same experimental parameters. The error of thrust $\langle T \rangle$ was estimated as a standard deviation $\delta(T)$.

The thrust-to-power ratio (TPR) was measured by dividing the thrust value $\langle T \rangle$ over the total power $\langle P \rangle$:

$$\langle TPR \rangle = \langle T \rangle / \langle P \rangle \tag{5}$$

The error for TPR was estimated using relative errors of the standard deviations for thrust $\delta(T)$ and total power $\delta(P)$:

$$\delta(TPR) = \langle TPR \rangle \left[\sqrt{\left(\delta(T)/\langle T \rangle\right)^2 + \left(\delta(P)/\langle P \rangle\right)^2} \right], \tag{6}$$

where $\langle TPR \rangle$, $\langle T \rangle$ and $\langle P \rangle$ are the average values of thrust-to-power ratio, thrust and power for the same experimental conditions.

Exhausting ion velocity

The exhausting ions velocity was determined by the time-of-flight method using experimental setup described in details in our previous study (*35*). Briefly, the thruster was fired with the plasma towards the two negatively-biased electrodes (first one is copper grid, the last one is stainless steel plate) placed at the distances s_{12} = 17 cm and s_{13} = 37 cm from the thruster exhaust. Once ions arrive at the electrode, they produce a pulsing current in the circuits of the each electrode. The ion velocity within single experiment *v* was estimated as:

$$v = \left[\left(s_{12}/t_{12}\right) + \left(s_{23}/t_{23}\right) + \left(s_{13}/t_{13}\right) \right] / 3, \tag{7}$$

where s_{12} is the distance between the thruster exhaust and the first electrode, s_{23} is the distance between electrodes, s_{13} is the distance between the thruster exhaust and the last electrode, t_{12}, t_{23} and t_{13} are respective time delays between maxima of the first-stage discharge current and ionic currents from each electrode. The final value of the ion velocity $\langle v \rangle$ was estimated as the average value of velocities *v* obtained within up to 6 experimental trials at the same experimental parameters. The error of ion velocity $\langle v \rangle$ was estimated as a standard deviation $\delta(v)$.

Specific impulse and energetic efficiency

Specific impulse $\langle I_{sp} \rangle$ was estimated from the ion velocity $\langle v \rangle$ as $\langle I_{sp} \rangle = \langle v \rangle / g_0$, where g_0 is the gravitational acceleration at the surface of the earth. The error of specific impulse $\delta(I_{sp})$ was estimated from the error of ion velocity $\delta(v)$ as $\delta(I_{sp}) = \delta(v)/g_0$.

Energetic efficiency η was estimated using the values of thrust, ion velocity and total power as $\eta = \langle T \rangle \langle v \rangle / \langle P \rangle$.

Mass flow rate

The mass flow rate was estimated according to the formula $\langle \dot{m} \rangle = \langle T \rangle / \langle v \rangle$ under assumption that all exhausting heavy particles are the ions which produce the thrust. The error in determination of mass flow rate was estimated as $\delta\left(\dot{m}\right) = \left\langle \dot{m} \right\rangle \left[\sqrt{\left(\delta(T)/\langle T \rangle\right)^2 + \left(\delta(v)/\langle v \rangle\right)^2} \right]$.

Total ion current measurement

Total ion current $I_i(t)$ was measured by placing the thruster inside a semispherical negatively-biased (-100 V) electrode (*25*, *35*). Then, the fractions of pulse-average total current $\langle I_i \rangle$ and charge $\langle Q_i \rangle$ of ions expelled by the thruster were estimated using the formulas:

$$\langle I_i \rangle \Big/ \left(\langle I_1 \rangle + \langle I_2 \rangle \right) = 100\% \times \left[\left(\frac{1}{\tau_i} \int_0^{\tau_i} I_i(t)dt \right) \Big/ \left(\frac{1}{\tau_1} \int_0^{\tau_1} I_1(t)dt + \frac{1}{\tau_2} \int_0^{\tau_2} I_2(t)dt \right) \right] \quad (8)$$

$$\langle Q_i \rangle \Big/ \left(\langle Q_1 \rangle + \langle Q_2 \rangle \right) = 100\% \times \left[\left(\int_0^{\tau_i} I_i(t)dt \right) \Big/ \left(\int_0^{\tau_1} I_1(t)dt + \int_0^{\tau_2} I_2(t)dt \right) \right] \quad (9)$$

where τ_i is the duration of the total ion current pulse, Q_1 and Q_2 are the charges of the first- and second-stage discharge currents. The error in determination of the mentioned ratios was estimated to the respective standard deviations.

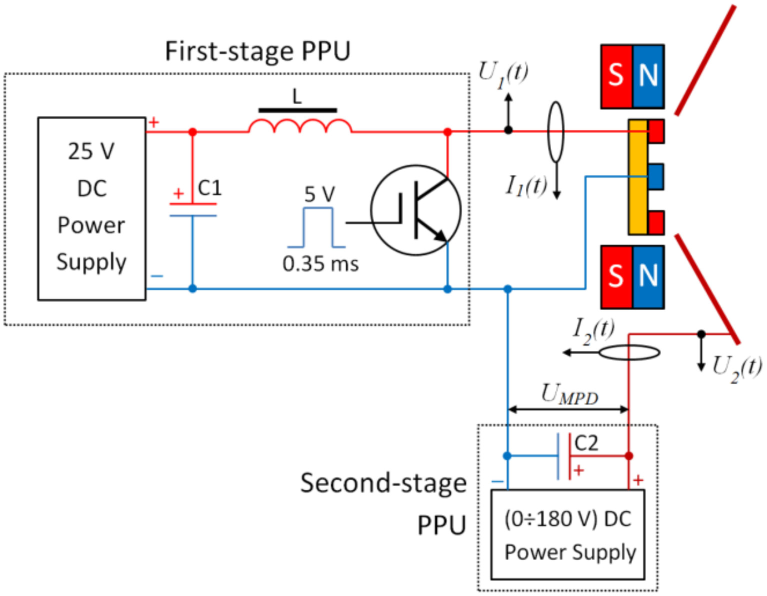

Fig. S1. Electrical circuit of the two-stage μCAT-MPD thruster.

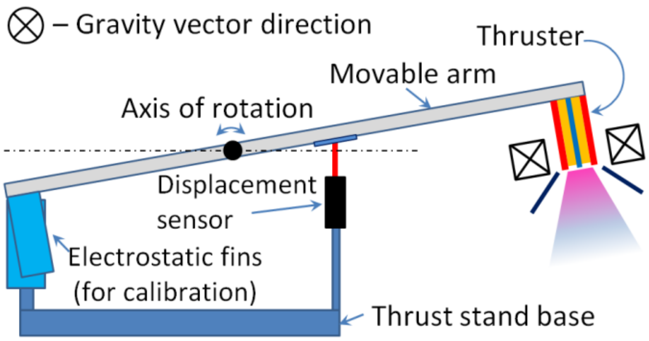

Fig. S2. Schematic view of the setup for the direct thrust measurement.

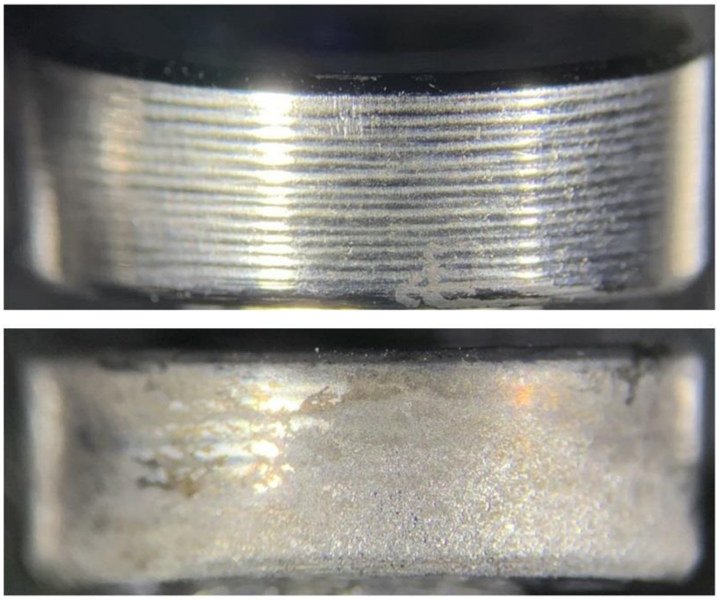

Fig. S3. Original (non-processed) images of the side surfaces of the cathodes after firing of the first stage only (upper image), and together with the second stage (lower image), within the single pulse.

REFERENCES AND NOTES

1. I. Levchenko, M. Keidar, J. Cantrell, Y.-L. Wu, H. Kuninaka, K. Bazaka, S. Xu, Explore space using swarms of tiny satellites. *Nature* **562**, 185–187 (2018).

2. I. Levchenko, S. Xu, G. Teel, D. Mariotti, M. L. R. Walker, M. Keidar, Recent progress and perspectives of space electric propulsion systems based on smart nanomaterials. *Nat. Commun.* **9**, 879 (2018).

3. I. Levchenko, S. Xu, S. Mazouffre, D. Lev, D. Pedrini, D. Goebel, L. Garrigues, F. Taccogna, K. Bazaka, Perspectives, frontiers, and new horizons for plasma-based space electric propulsion. *Phys. Plasmas* **27**, 020601 (2020).

4. Xenon Market Size, Share & Covid-19 impact analysis, by type (N3, N4.5, and N5) by application (imaging and lighting, satellite, electronics & semiconductors, medical, and others) and regional forecast, 2021-2028 (Chemical & Materials, 2021); www.fortunebusinessinsights.com/xenon-market-101965 [accessed 29 April 2022].

5. J. Son, J. Kwon, S. Kim, Y. Lv, J. Yu, J.-Y. Lee, H. Ryu, K. Watanabe, T. Taniguchi, R. Garrido-Menacho, N. Mason, E. Ertekin, P. Y. Huang, G.-H. Lee, A. M. van der Zande, Atomically precise graphene etch stops for three dimensional integrated systems from two dimensional material heterostructures. *Nat. Commun.* **9**, 3988 (2018).

6. A. E. Neice, M. H. Zornow, Xenon anaesthesia for all, or only a select few? *Anaesthesia* **71**, 1267–1272 (2016).

7. D. Rafalskyi, J. Martínez Martínez, L. Habl, E. Zorzoli Rossi, P. Proynov, A. Boré, T. Baret, A. Poyet, T. Lafleur, S. Dudin, A. Aanesland, In-orbit demonstration of an iodine electric propulsion system. *Nature* **599**, 411–415 (2021).

8. S. Hurley, G. Teel, J. Lukas, S. Haque, M. Keidar, C. Dinelli, J. Kang, Thruster subsystem for the United States Naval Academy's (USNA) Ballistically Reinforced Communication Satellite (BRICSat-P). *Trans. Japan Soc. Aeronaut. Space Sci. Aerosp. Technol. Japan* **14**, Pb157–Pb163 (2016).

9. J.-P. Park, S. Park, K. Lee, H. J. Oh, K. Y. Choi, Y. B. Song, J.-C. Yim, E. Lee, S.-H. Hwang, S. W. Kim, S. J. Kang, M.-S. Kim, S. Jin, S. H. Lee, S. H. Kwon, D. S. Lee, W.-H. Cho, J.-H. Park, S.-W. Yeo, J.-W. Seo, K. B. Lee, S.-H. Lee, J.-H. Yang, G. N. Kim, J. Lee, Y. W. Kim, T.-H. Kim, Cubesat development for CANYVAL-X mission, paper presented at the SpaceOps Conferences Daejeon, Korea, 16 to 20 May 2016.

10. J. T. King, J. Kolbeck, J. S. Kang, M. Sanders, M. Keidar, Performance analysis of nano-sat scale μCAT electric propulsion for 3U CubeSat attitude control. *Acta Astronaut.* **178** 722–732 (2021).

11. D. B. Zolotukhin, K. P. Daniels, L. Brieda, M. Keidar, Onset of the magnetized arc and its effect on the momentum of a low-power two-stage pulsed magneto-plasma-dynamic thruster. *Phys. Rev. E* **102**, 021203 (2020).

12. I. I. Beilis, *Plasma and Spot Phenomena in Electrical Arcs* (Springer Nature, 2020), vol. 1 and 2.

13. I. I. Beilis, Vacuum arc cathode spot theory: History and evolution of the mechanisms. *IEEE Trans. Plasma Sci.* **47**, 3412–3433 (2019).

14. I. I. Beilis, Y. Koulik, R. L. Boxman, D. Arbilly, Thin film deposition using a plasma source with a hot refractory anode vacuum arc. *J. Mater. Sci.* **45**, 6325–6331 (2010).

15. C. W. Kimblin, Erosion and ionization in the cathode spot regions of vacuum arcs. *J. Appl. Phys.* **44**, 3074–3081 (1973).

16. A. Anders, E. M. Oks, G. Yu. Yushkov, K. P. Savkin, I. G. Brown, A. G. Nikolaev, Measurements of the total ion flux from vacuum arc cathode spots. *IEEE Trans. Plasma Sci.* **33**, 1532–1536 (2005).

17. J. E. Daalder, Erosion and the origin of charged and neutral species in vacuum arcs. *J. Phys. D Appl. Phys.* **8**, 1647–1659 (1975).

18. S. K. Sethuraman, P. A. Chatterton, A study of the erosion rate of vacuum arcs in a transverse magnetic field. *J. Nucl. Mater.* **111-112** 510–516 (1982).

19. T. Utsumi, J. H. English, Study of electrode products emitted by vacuum arcs in form of molten metal particles. *J. Appl. Phys.* **46**, 126–131 (1975).

20. S. Shalev, S. Goldsmith, R. L. Boxman, In situ determination of macroparticle velocities in a copper vacuum arc. *IEEE Trans. Plasma Sci.* **11**, 146–151 (1983).

21. S. Shalev, R. L. Boxman, S. Goldsmith, Velocities and emission rates of cathode-produced molybdenum macroparticles in a vacuum arc. *J. Appl. Phys.* **58**, 2503–2507 (1985).

22. E. Byon, A. Anders, Ion energy distribution functions of vacuum arc plasmas. *J. Appl. Phys.* **93**, 1899–1906 (2003).

23. A. Anders, G. Y. Yushkov, Ion flux from vacuum arc cathode spots in the absence and presence of a magnetic field. *J. Appl. Phys.* **91**, 4824–4832 (2002).

24. F. F. Chen, *Introduction to Plasma Physics and Controlled Fusion* (Plenum, 1984).

25. D. B. Zolotukhin, K. P. Daniels, S. R. P. Bandaru, M. Keidar, Magnetoplasmadynamic two-stage micro-cathode arc thruster for CubeSats. *Plasma Sources Sci. Technol.* **28**, 105001 (2019).

26. J. Lun, C. Law, Direct thrust measurement stand with improved operation and force calibration technique for performance testing of pulsed micro-thrusters. *Meas. Sci. Technol.* **25**, 095009 (2014).

27. I. Kronhaus, K. Schilling, M. Pietzka, J. Schein, Simple orbit and attitude control using vacuum arc thrusters for picosatellites. *J. Spacecr. Rockets* **51**, 2008–2015 (2014).

28. I. Kronhaus, M. Laterza, Y. Maor, Inline screw feeding vacuum arc thruster. *Rev. Sci. Instrum.* **88**, 043505 (2017).

29. D. B. Zolotukhin, M. Keidar, Optimization of discharge triggering in micro-cathode vacuum arc thruster for CubeSats. *Plasma Sources Sci. Technol.* **27**, 074001 (2018).

30. A. Anders, J. Schein, N. Qi, Pulsed vacuum-arc ion source operated with a “triggerless” arc initiation method. *Rev. Sci. Instrum.* **71**, 827–829 (2000).

31. J. M. Lafferty, *Vacuum Arcs: Theory and Applications* (John Wiley & Sons, 1980).

32. K. Holste, P. Dietz, S. Scharmann, K. Keil, T. Henning, D. Zschätzsch, M. Reitemeyer, B. Nauschütt, F. Kiefer, F. Kunze, J. Zorn, C. Heiliger, N. Joshi, U. Probst, R. Thüringer, C. Volkmar, D. Packan, S. Peterschmitt, K.-T. Brinkmann, H.-G. Zaunick, M. H. Thoma, M. Kretschmer, H. J. Leiter, S. Schippers, K. Hannemann, P. J. Klar, Ion thrusters for electric propulsion: Scientific issues developing a niche technology into a game changer, *Rev. Sci. Instrum.* **91** 061101 (2020).

33. M. Peukert, B. Wollenhaupt, "OHB-System's view on electric propulsion needs," in Presentation, EPIC Workshop, Brussels, Belgium, 2014.

34. B. Wollenhaupt, M. Peukert, R. Gabrielli, Comparison of mission needs with available electric propulsion technologies, paper presented at the Space Propulsion Conference, Rome, Italy, 2016.

35. D. B. Zolotukhin, A. V. Tyunkov, Yu. G. Yushkov, E. M. Oks, M. Keidar. Improvement of micro-cathode arc thruster lifetime by deposition of boron-containing coating, *J. Propuls. Power* **36**, 744–751 (2020).
